# From shifting cultivation to teak plantation: effect on overland flow and sediment yield in a montane tropical catchment

**DOI:** 10.1038/s41598-017-04385-2

**Published:** 2017-06-21

**Authors:** Olivier Ribolzi, Olivier Evrard, Sylvain Huon, Anneke de Rouw, Norbert Silvera, Keo Oudone Latsachack, Bounsamai Soulileuth, Irène Lefèvre, Alain Pierret, Guillaume Lacombe, Oloth Sengtaheuanghoung, Christian Valentin

**Affiliations:** 1 Institut de Recherche pour le Développement (IRD), Géosciences Environnement Toulouse (GET), UMR 5563 (CNRS-UPS-IRD), Toulouse, France; 2 Laboratoire des Sciences du Climat et de l’Environnement (LSCE/IPSL), Unité Mixte de Recherche 8212 (CEA-CNRS-UVSQ), Université Paris-Saclay, Gif-sur-Yvette, France; 3 Université Pierre et Marie Curie (UPMC), UMR 7618 iEES (UPMC-CNRS-IRD-INRA-Université Paris 7-UPEC), Paris, France; 4 Institut de Recherche pour le Développement (IRD), UMR 7618 iEES (UPMC-CNRS-IRD-INRA-Université Paris 7-UPEC), Paris, France; 5 Institut de Recherche pour le Développement (IRD), iEES-Paris, Centre IRD France-Nord, Bondy, France; 6 Institut de Recherche pour le Développement (IRD), Department of Agricultural Land Management (DALaM), Vientiane, Lao PDR; 7 International Water Management Institute (IWMI), Southeast Asia Regional Office, Vientiane, Lao PDR; 8 Department of Agricultural Land Management (DALaM), Ministry of Agriculture and Forestry, Vientiane, Lao PDR

## Abstract

Soil erosion supplies large quantities of sediments to rivers of Southeastern Asia. It reduces soil fertility of agro-ecosystems located on hillslopes, and it degrades, downstream, water resource quality and leads to the siltation of reservoirs. An increase in the surface area covered with commercial perennial monocultures such as teak plantations is currently observed at the expanse of traditional slash-and-burn cultivation systems in steep montane environments of these regions. The impacts of land-use change on the hydrological response and sediment yields have been investigated in a representative catchment of Laos monitored for 13 years. After the gradual conversion of rice-based shifting cultivation to teak plantation-based systems, overland flow contribution to stream flow increased from 16 to 31% and sediment yield raised from 98 to 609 Mg km^−2^. This result is explained by the higher kinetic energy of raindrops falling from the canopy, the virtual absence of understorey vegetation cover to dissipate drop energy and the formation of an impermeable surface crust accelerating the formation and concentration of overland flow. The 25-to-50% lower ^137^Cs activities measured in soils collected under mature teak plantations compared to soils under other land uses illustrate the severity of soil erosion processes occurring in teak plantations.

## Introduction

Soil erosion is exacerbated by the intensification of agriculture in montane steep cultivated catchments of Southeastern Asia, where it delivers significant quantities of sediment to rivers^1^. This excessive sediment supply leads to water reservoir siltation^2^ and transports particle-bound contaminants towards downstream areas^3–5^. Among these pollutants, there are growing concerns regarding the transport of persistent pathogenic bacteria with sediment and their development in the streambed of tropical catchments^6^, where the use of unclean stream water is associated with a high number of fatal and debilitating diseases^7^.

The impact of land-use change on soil erosion has been investigated in various regions of Southeastern Asia^8^, although most studies were restricted to the plot and hillslope scales^9, 10^. The main factors known to control soil erosion at the plot scale^1, 11^ are rainfall, overland flow and surface characteristics (e.g. land use, vegetation cover, slope gradient, slope length, surface crusting). At this scale, inter-rill erosion processes dominate and mainly detach and mobilize soil surface particles. Then, at the hillslope scale, inter-rill erosion is combined with overland flow, which may concentrate and form rills and gullies, thereby removing and transporting additional particles originating from the soil subsurface^12–14^. Finally, at the catchment scale, the dominance of overland flow as the main supply of sediment export at the outlet needs to be more thoroughly investigated, as this phenomenon is in competition with other processes (e.g. reinfiltration and sedimentation, subsurface flow contribution). Although the direct measurement of overland flow is possible at the plot and hillslope scales, it is difficult to carry out similar measurements at the catchment scale. At this larger scale, indirect tracer-based approaches provide an effective method to calculate the respective contributions of subsurface and surface flow. Typically, water mixing models using geochemical properties (e.g. electric conductivity, δ^18^O) are applied to quantify these contributions^15, 16^.

In forested catchments, subsurface contributions were shown to provide the dominant contribution to streamflow^17, 18^, whereas surface overland flow was identified as the main supply in cultivated catchments where an almost impermeable crust may be formed at the soil surface^15, 19, 20^. Most land-use change studies investigated the conversion of forests into cropland, showing the associated increase in soil erosion^21, 22^. To our knowledge, only few studies have been conducted on the conversion of annual crops into commercial tree monoculture plantations on the steep hillslopes of humid tropics although this rapid land-use change is affecting extensive areas across this region^23^. The main objective associated with the reforestation of degraded land is often to restore the soil infiltration capacity and to limit soil erosion. However, it was shown that this reforestation may also increase plant water uptake and consequently reduce river baseflow^24, 25^.

**Figure 1:**
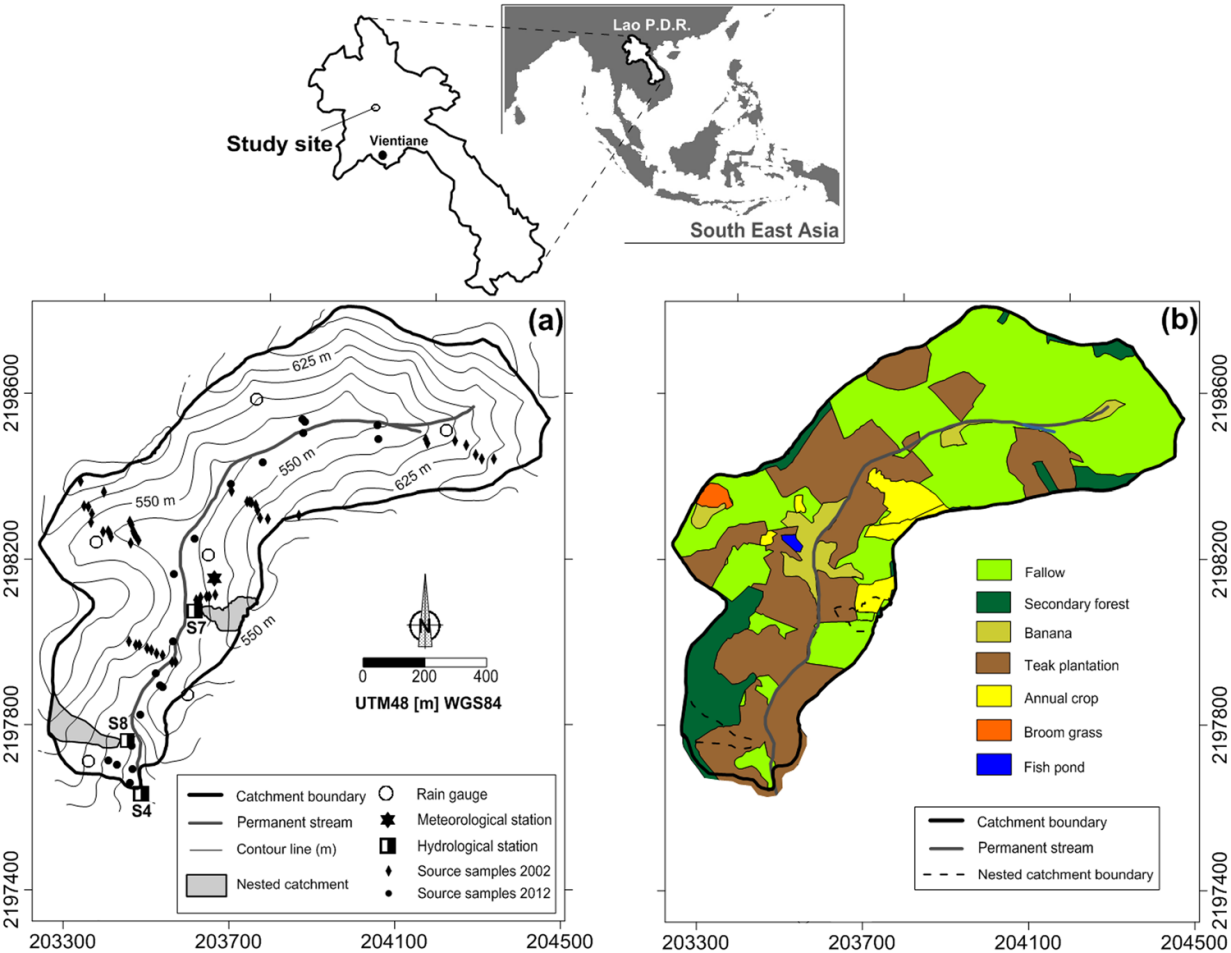
Monitoring devices and sampling sites in the Houay Pano catchment. This original map was created using Surfer 13.2.438 from Golden Software, LLC.

To investigate the impact of land-use change on sediment yield, the monitoring of paired catchments is often conducted in nearby sites where contrasted land uses prevail while biophysical characteristics are as similar as possible. This synchronic approach suffers from several limitations because of the difficulty to select two perfectly identical catchments^26^. In contrast, this approach is easier to conduct in similar zero-order headwater catchments (i.e. hollow valleys referred to as S7 and S8) with similar soil characteristics although different land uses. In addition to this synchronic approach, we applied a diachronic investigation in the larger catchment affected by land use change and draining these hollow valleys. The 0.6-km² Houay Pano catchment (Fig. 1), located in Northern Laos, is representative of areas that experienced large-scale afforestation with commercial teak tree (*Tectona grandis* L.f.) plantations at the expense of traditional slash-and-burn (i.e. shifting) cultivation. Discharge and sediment yield were monitored for 13 years in this experimental catchment.

The contributions of surface (i.e. overland flow) and sub-surface flow (i.e. groundwater) to river water at the catchment outlet were assessed by measuring the river water electrical conductivity. This technique is easier to implement than the more conventional method based on δ^18^O, and both techniques were found to provide similar results^15^. The contributions of surface and sub-surface soil sources to stream sediment exports at the catchment outlet were discriminated by measuring the activity of Caesium-137 (^137^Cs) emitted by thermonuclear bomb tests in the 1960s in the sediment transiting the river^27^. This radioisotope characterised by a half-life of 30 years shows highly contrasting activities between the surface of cultivated soils exposed to atmospheric fallout and gully/channel bank material sheltered from this fallout^28–30^.

## Results

### Land-use change

**Figure 2:**
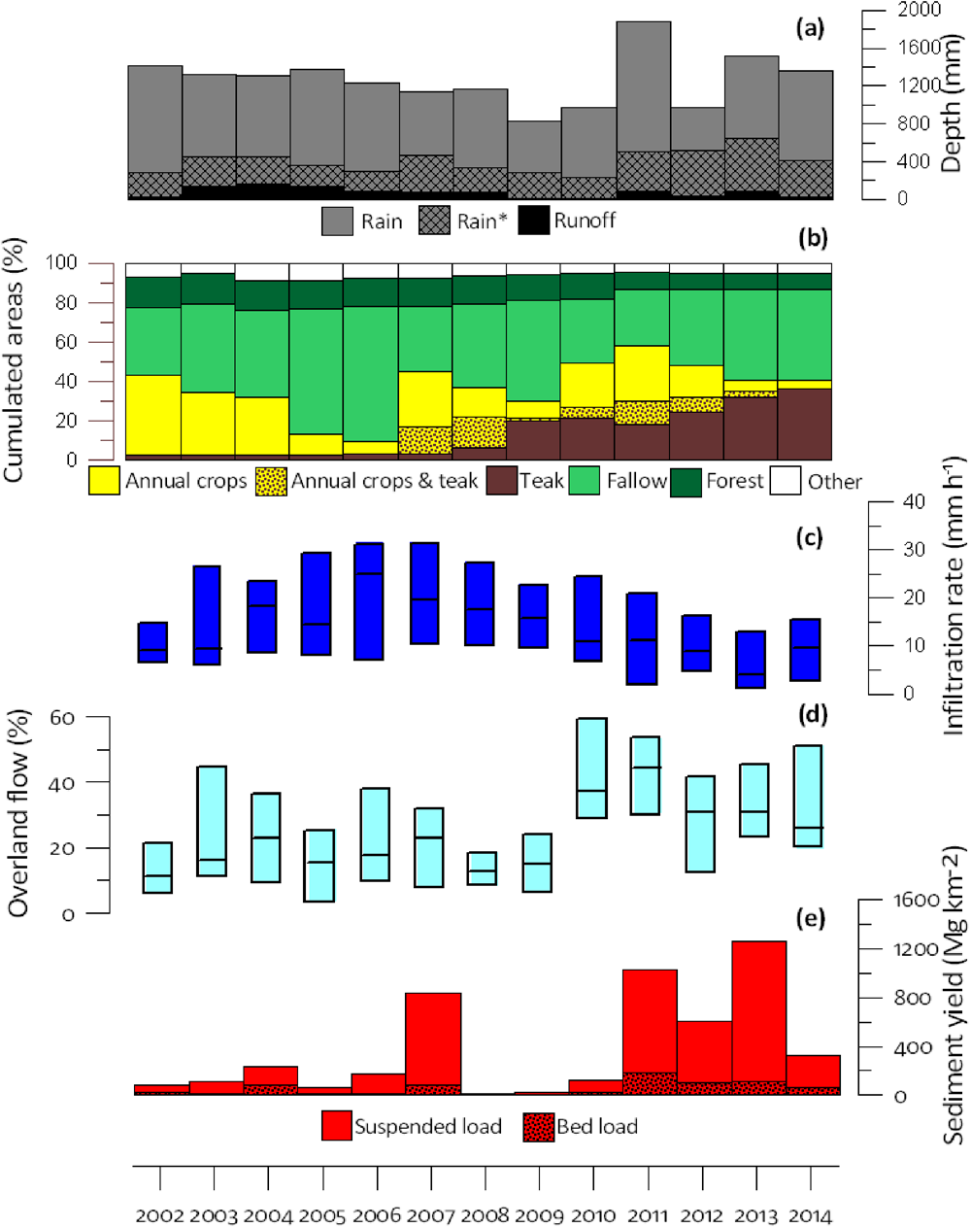
Annual hydro-sedimentary and land-use changes in Houay Pano catchment: (**a**) Total rainfall depth (Rain), flood-triggering rainfall depth (Rain_F) and total stream flow depth (Runoff); (**b**) Areal percentages of the catchment covered by the main land uses (i.e. annual crops; annual crops associated with one-or-two-years-old teak trees; teak plantations; fallow; secondary forest and other land uses including gardens and banana); (**c**) Box plots (25^th^ and 75^th^ percentiles and median) of infiltration rates computed for each flood event; (**d**) Box plots of overland flow contribution to total depth of flood events estimated using tracer-based hydrograph separation; (**e**) Annual sediment yield including suspended load and bed load contributions. This graph was created using Grapher 4.0 from Golden Software, LLC.

The proportion of secondary forests in the catchment decreased from 16% (2002) to 8% (2014) (Fig. 2b). During this period, the variations in the respective proportions of fallow (29–69%) and annual crops (4–42%) were negatively correlated and they showed large inter-annual variations, reflecting the management strategies of replacing fallows with annual crops through slash-and-burn, followed by the regrowth of vegetation in the temporarily abandoned cultivated fields. This entire cycle may take 2 to 6 years (Fig. 2b). Until 2007, the percentage of catchment area covered with teak plantations (Teak) remained low and did not exceed 4%. From 2008, it continuously increased, almost linearly, to reach a maximum of 36% of the catchment area in 2014 (Fig. 2b), illustrating the transition from an annual crop-based system towards an organisation dominated by tree plantations. Accordingly, the monitoring period was divided into a first sub-period (2002–2007) characterized by the alternation between upland rice (*Oryza sativa* L.), Job’s tear (*Coix lacryma-jobi* L.) annual crops and bush fallow, in addition to sparse teak plantations, and a second sub-period (2008–2014) characterised by the concomitant expansion of teak plantations and the reduction of annual crops. During the last five years of this second sub-period, most of the teak plantations were more than 3 years old and characterised by limited understorey vegetation cover, which may enhance soil degradation. In 2014, land use greatly differed between the two hollow valleys investigated in details (Fig. 1b), with a dominant contribution of typical shifting cultivation landscape mosaics at S7 compared to a major contribution of teak plantations at S8.

### Rainfall and runoff

**Figure 3:**
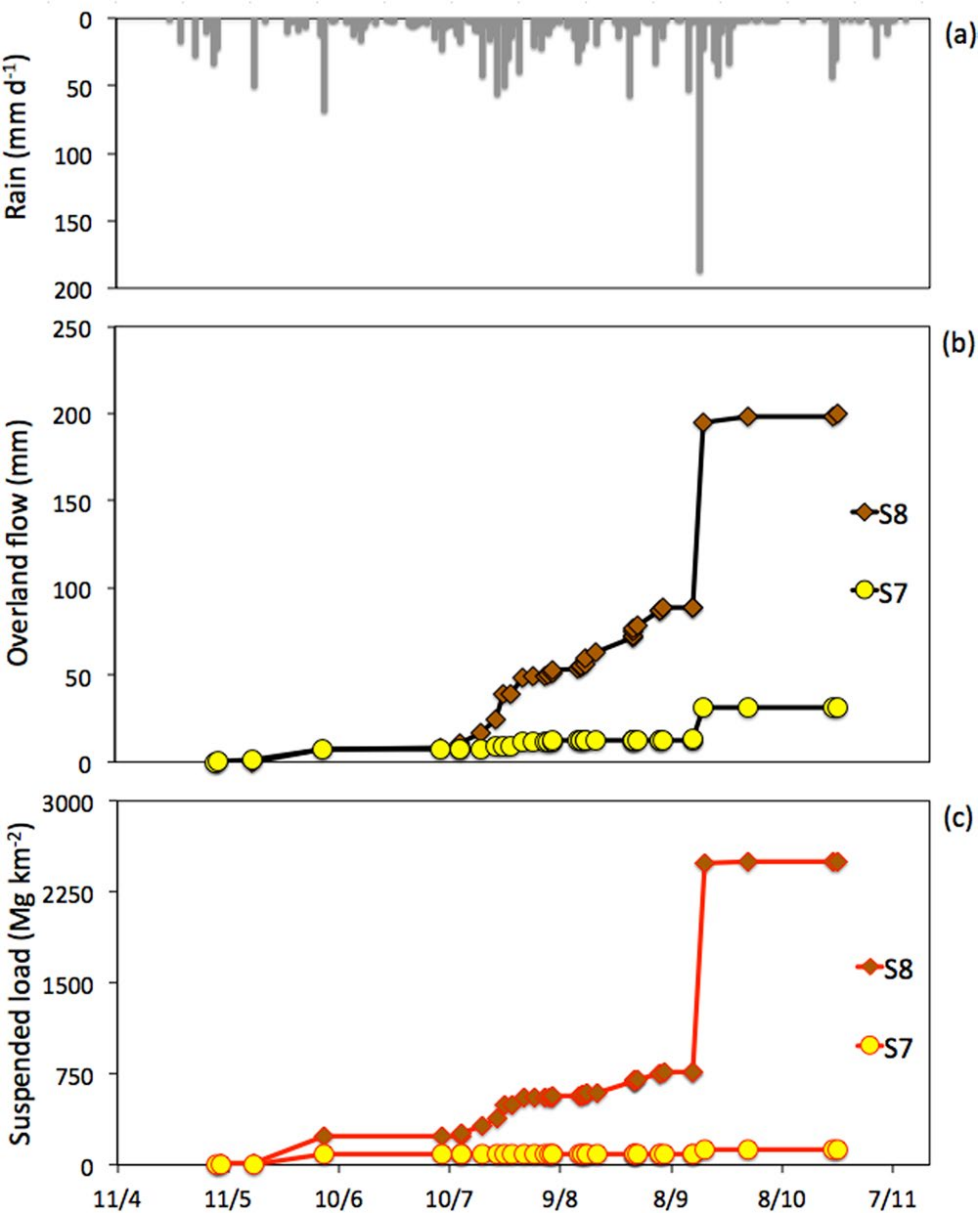
(**a**) Daily rainfall depth, (**b**) cumulated suspended sediment yield and, (**c**) overland flow measured in 2014 at the hydrological station installed at the outlet of two zero-order headwater subcatchments (i.e. ephemeral flow): S7 (dominance of traditional shifting cultivation mosaics); S8 (dominance of teak plantations). This graph was created using Excel 14.0 Software.

During the 2002–2014 monitoring period of the Houay Pano catchment, annual rainfall depth (Rain) varied between 978 mm (2012) and 1884 mm (2011), with a mean of 1271 mm (SD: 271 mm, CV: 20%) (Fig. 2a). These values are similar to those recorded at the closest reference weather station located in Luang Prabang (58-year long-term mean: 1302 mm; SD: 364 mm; CV: 28%). Only 32% of the rainfall events generated stormflow (i.e. total stream flow during a flood that is calculated when summing surface and subsurface contributions). The mean annual depth corresponding to these flood-triggering rainfall events (Rain_F) was 404 mm (SD: 118 mm; CV: 28%). Annual runoff showed large inter-annual variations (SD: 52 mm; CV: 67%; mean: 77 mm; range: 13–174 mm) (Fig. 2a). In 2014, runoff from the hollow valleys strongly differed (Friedman non-parametric test for paired samples [α = 0.0001] p < 0.0001) between S7 (32 mm) and S8 (200 mm; Fig. 3b).

### Infiltration rate, overland flow and sediment yield

The annual median values of infiltration rates calculated for each flood event at the catchment scale (InfRate) strongly varied, between 4 and 25 mm h^−1^ depending on years (Fig. 2c). It first increased from 9 mm h^−1^ to 20 mm h^−1^ during the first sub-period (i.e. 2002–2007), and then decreased gradually from 18 to 4 mm h^−1^ during the second sub-period (i.e. 2008–2014). Although it is characterised by large inter-annual variations, the annual median of overland flow contribution to the total stream flow during floods (F_OLF_) was significantly higher during the last five years of the second study period (2010–2014; inter-annual median: 31%) when soils of the teak plantations were already degraded and characterised by sparse understorey vegetation cover (Mann-Whitney bilateral non-parametric test [α = 0.01] p = 0.004) compared to the first sub-period (2002–2009; inter-annual median: 16%) (Fig. 2d). Similarly, annual sediment yields (SY) exhibited large inter-annual fluctuations between 10 Mg km^−^² and 1,260 Mg km^−^², with a significant increase (Mann-Whitney bilateral non-parametric test [α = 0.05] p = 0.003) from the first (2002–2009; inter-median: 98 Mg km^−2^) to the second (2010–2014; inter-median: 609 Mg km^−2^) sub-periods (Fig. 2e). Bed load contribution to these total annual losses fluctuated between 9 and 42%, with similar averages during the first and second sub-periods (mean of 16% and 15%, respectively). In the hollow valleys in 2014, cumulated sediment yield remained negligible (Friedman non-parametric test for paired samples [α = 0.0001] p < 0.0001) at S7 (127 Mg km^−2^), whereas it was very high at S8 (2499 Mg km^−2^; Fig. 3c).

**Figure 4:**
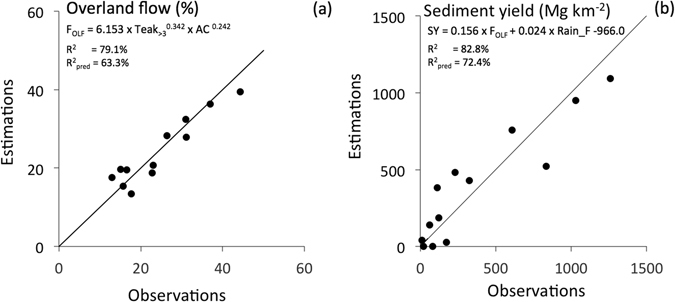
Statistical relationships (data collected at the S4 monitoring station) between: (**a**) median overland flow contribution to stream flow during storm events (F_OLF_, %) and the two explanatory variables: areal percentage of teak monocultures during year *n*-1 (Teak_>3_, %) and areal percentage of annual crops cultivated alone or in combination with one-or-two-years-old teak trees (AC, %); (**b**) median sediment yield (SY, Mg km^−2^) and the two explaining variables F_OLF_ and annual flood-triggering rainfall depth (Rain_F, mm). This graph was created using Excel 14.0 Software.

A correlation analysis was performed to investigate the relationships between the catchment hydrological behaviour, land uses and sediment yields. The main objective of this analysis was to evaluate how the transition from an annual crop-based system towards a tree plantation-based system affected the hydro-sedimentary response of the catchment. We evaluated the significance of the correlation between several variables reported in Table 1. Among these parameters, the areal percentage of more than 3-year-old teak tree monocultures (Teak_>3_) was considered. Teak_>3_ was used in order to exclude the youngest plantations which were not yet submitted to burning (Fig. 4a), and in which soils were not yet degraded.

**Table 1:** Correlation matrix (Pearson correlation coefficients; *p-value < 0.05, **p-value < 0.01, ***p-value < 0.001) between the following variables: annual rainfall depth (Rain); flood-triggering annual rainfall depth (Rain_F); annual overland flow depth (OLF); annual subsurface flow depth (SSF); annual median of overland flow contribution to total depth of each flood event (F_OLF_); annual median of subsurface flow contribution to total depth of each flood event (F_SSF_); annual median of event infiltration rates at the catchment scale (InfRate); annual suspended load (SL); annual bed load (BL); Total sediment yield (SY, i.e. SL + BL); areal percentage of teak tree monocultures (Teak); areal percentage of teak tree monocultures of more than 3-year-old (Teak_>3_); areal percentage of annual crops cultivated alone or in combination with one-or-two-years-old teak (AC); areal percentage of fallow and secondary forest (Fw&Fo).

	Rain mm	Rain_F mm	OLF mm	SSF mm	F_OLF_ %	F_SSF_ %	InfRate mm h^−1^	SL Mg km^−2^	BL Mg km^−2^	SY Mg km^−2^	Teak %	Teak_>3_%	AC %
Rain	1												
Rain_F	0.55	1											
OLF	0.69*	0.49	1										
SSF	0.32	0.18	0.94	1									
F_OLF_	0.43	0.39	0.17	-0.45	1								
F_SSF_	−0.42	−0.39	−0.17	0.46	−1***	1							
InfRate	−0.25	−0.55	0.02	0.28	−0.52	0.52	1						
SL	0.52	0.83***	0.26	−0.15	0.62*	−0.62*	−0.44	1					
BL	0.62*	0.75**	0.45	−0.16	0.81**	−0.81**	−0.41	0.82**	1				
SY	0.55	0.84***	0.29	−0.16	0.66*	−0.66*	−0.44	0.99***	0.86***	1			
Teak	0.05	0.28	−0.37	−0.69*	0.55	0.55	−0.71**	0.40	0.39	0.40	1		
Teak_>3_	0.30	0.35	−0.18	−0.60*	0.73**	−0.73**	−0.72**	0.49	0.55	0.51	0.93***	1	
AC	0.14	0.17	0.37	0.07	0.29	−0.29	0.07	0.19	0.34	0.21	−0.40	−0.24	1
Fw&Fo	−0.18	−0.42	−0.05	0.49	−0.75**	−0.75**	0.52	−0.53	−0.68*	−0.56	−0.45	−0.55	−0.64*

Contrary to the total annual overland flow depth (OLF), which is correlated to Rain (*p*-value < 0.05;), F_OLF_ is not. In contrast, F_OLF_ is positively correlated to Teak_>3_ (*p*-value < 0.05; Table 1) and negatively correlated to the sum of fallow and forest areas (Fw&Fo) (*p*-value < 0.05), and it provides therefore a better proxy of land use change. This is also confirmed by the regression analysis with a positive correlation with annual crops cultivated alone or in combination with one-or-two-years-old teak (AC) (Fig. 4a). InfRate is very significantly and negatively correlated to Teak and Teak_>3_ (*p*-value < 0.01) (Table 1).

Sediment yield was strongly correlated to the annual suspended load (SL) (*p*-value < 0.001), which is the main export route for soil particles (SL account for 80 and 78% of SY, on average, during the first and the second sub-periods, respectively). Annual bed load (BL) is also well correlated to SL (*p*-value < 0.01). Interestingly, SY (i.e. BL + SL) is correlated to Rain_F (*p*-value < 0.001, < 0.01 and < 0.001 respectively; Table 1) and F_OLF_ (*p*-value < 0.05, 0.01 and 0.05 respectively), which agrees with the regression equation (Fig. 4b). A negative correlation was observed between the areal percentage of Fw&Fo and soil losses. However, it was significant for BL only (*p*-value < 0.05) (Table 1).

Multiple log-linear and linear regressions were conducted to identify explanatory variables that best predicted the inter-annual variations of F_OLF_ and SY. All the variables listed in Table 1 were considered in this analysis. A best sub-set of two explanatory variables was selected in each regression: Teak_>3_ and the areal percentage of annual crops cultivated alone or in combination with one-or-two-years-old teak (AC) for F_OLF_ (Fig. 3a); Rain_F and F_OLF_ for SY (Fig. 4b). While the regression model predicting SY was linear, a stronger log-linear relationship was observed between F_OLF_ and its explanatory variables, reflecting the typically skewed and non-normal statistical distributions of hydrological variables. F_OLF_ and SY were statistically predicted using the equations presented in Fig. 4.

### Soil and sediment signatures

**Figure 5:**
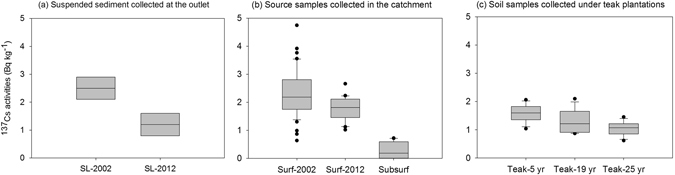
^137^Cs activities measured in (**a**) annual composite sediment samples in 2002 (SL-2002) and 2012 (SL-2012) at the catchment outlet (S4 monitoring station); (**b**) potential sources of sediment (surface of cultivated soils sampled between 2000–2005 [Surf-2002]; surface of cultivated soils sampled in 2012 [Surf-2012]; subsurface samples collected in 2012 in gullies and channel banks [Subsurf]; (**c**) Surface soil under teak plantations of different maturity levels: 5-years old plantation [Teak-5 yr]; 19-years old plantation [Teak-19 yr]; 25-years old plantation [Teak-25 yr]). This original graph was created using SigmaPlot 12 software.

The ^137^Cs signature in the composite sample of suspended sediment representative of the annual sediment export from the catchment decreased by more than 50% during the study period (from 2.5 ± 0.4 Bq kg^−1^ in 2002, to 1.2 ± 0.4 Bq kg^−1^ in 2012; Fig. 5a). In potential sediment sources, ^137^Cs activities varied between subsurface soils (0.4 ± 0.3 Bq kg^−1^), surface soils collected in 2002 (2.3 ± 0.8 Bq kg^−1^) and surface soils collected in 2012 (1.8 ± 0.4 Bq kg^−1^; Fig. 5b). These values were used to discriminate material originating from the soil surface and sub-surface in the collected samples, with a high confidence (*p*-value < 0.01; Table 2). Despite a decrease in ^137^Cs activities recorded in cultivated soils between 2002 and 2012 (*p*-value < 0.05), which likely reflects the high intensity of erosion that occurred in cropland during the last decade, ^137^Cs activities found in surface soils collected in 2002 and 2012 remained significantly higher than those found in subsurface material (*p*-value < 0.01). Interestingly, surface soils under young (5-years old) teak plantations were characterized by activities (1.6 ± 0.3 Bq kg^−1^) similar to those measured in surface soils in 2012 (1.8 ± 0.4 Bq kg^−1^, Table 2) whereas surface soils collected under the most mature (19–25 years old) teak plantations showed significantly different signatures (1.1–1.3 ± 0.2–0.3 Bq kg^−1^) compared to 2012 surface measurements (1.8 ± 0.4 Bq kg^−1^, *p*-value < 0.05; Table 2) and subsurface (0.4 ± 0.3 Bq kg^−1^, *p*-value < 0.01; Table 2) material, corresponding to an ‘intermediate’ signature between surface and subsurface sources.

**Table 2:** Results of the Tukey−Kramer Honestly Significant Difference (HSV) test conducted on ^137^Cs activities measured in the soil and sediment sample groups shown in Fig. 5.

	SL-2002	Surf-2002	Surf-2012	Teak-5 yrs	Teak-19 yrs	Teak-25 yrs	Subsurf	SL-2012
SL-2002		insignificant	insignificant	insignificant	*p < 0.05	**p < 0.01	**p < 0.01	insignificant
Surf-2002	insignificant		*p < 0.05	**p < 0.01	**p < 0.01	**p < 0.01	**p < 0.01	*p < 0.05
Surf-2012	insignificant	*p < 0.05		insignificant	insignificant	*p < 0.05	**p < 0.01	insignificant
Teak-5 yrs	insignificant	**p < 0.01	insignificant		insignificant	insignificant	**p < 0.01	insignificant
Teak-19 yrs	*p < 0.05	**p < 0.01	insignificant	insignificant		insignificant	**p < 0.01	insignificant
Teak-25 yrs	**p < 0.01	**p < 0.01	*p < 0.05	insignificant	insignificant		**p < 0.01	insignificant
Subsurf	**p < 0.01	**p < 0.01	**p < 0.01	**p < 0.01	**p < 0.01	**p < 0.01		insignificant
SL-2012	insignificant	*p < 0.05	insignificant	insignificant	insignificant	insignificant	insignificant	

SL = suspended sediment load, Surf = soil surface (<5 cm), Subsurf = soil subsurface (gullies and channel banks).

## Discussion

### Impact of land-use change on infiltration rates and overland flow

In contrast to teak plantations^9^, fallows are characterised by higher infiltration rates associated with a higher biological activity maintaining greater porosity and lower surface crusting^31^. Several vegetation strata, including understorey, are observed in fallows. By dissipating the kinetic energy of raindrops, they reduce the soil surface crusting. In contrast, under teak canopies where large leaves concentrate rainfall, heavier rain drops fall from higher interception heights, impacting the soil with a greater kinetic energy^30^, clogging the soil surface porosity and disturbing the soil surface structure, both processes contributing to crusting and the associated reduction in infiltration rates. Consequently, the production of overland flow increases and detaches more soil particles by sheet flow.

**Figure 6:**
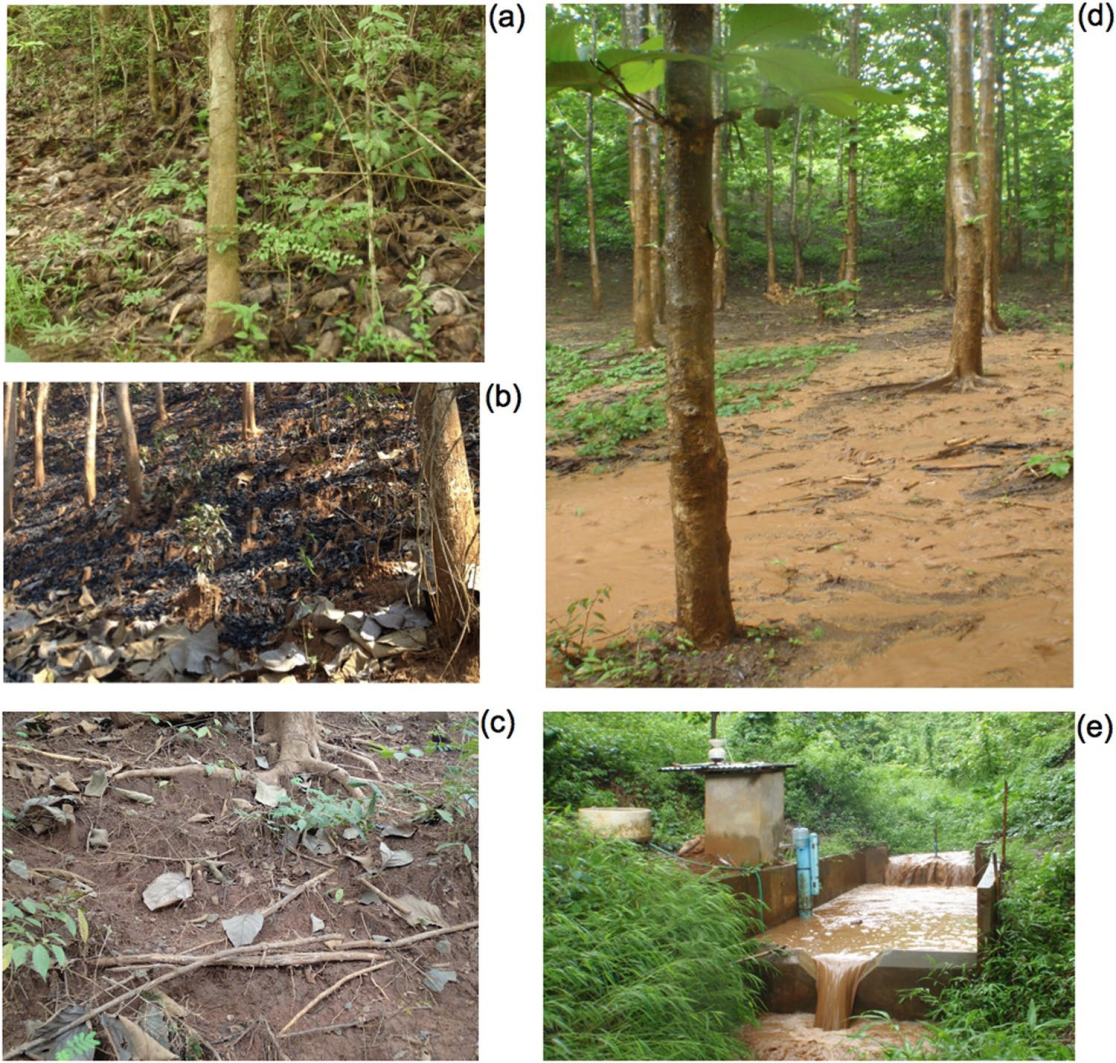
Pictures taken in the Houay Pano catchment: (**a**) teak understorey in natural conditions (vegetation and litter are present); (**b**) teak understorey after a fire (litter and vegetation virtually cleared); (**c**) degraded soil surface with erosion crust and exposed teak roots; (**d**) sheet overland flow under a teak plantation during a rainfall event; (**e**) the hydrological station (so-called S4 station) equipped with a sediment trap for the quantification of bed load (larger blue PVC tube), an automatic sampler for the estimation of suspended solid load, a water level recorder and V-nutch weir to monitor the stream discharge, and a probe for measuring the electrical conductivity of stream water (in the smaller blue PVC tube).

The contribution of surface overland flow to the total stream flow increased during the second sub-period. This evolution reflects a change in the hydrological behaviour of the catchment, which coincides with the increase of the teak cover at the expense of fallow and annual crops. Until it is two-year old, teak is generally interspersed with annual crops (Fig. 2b). From the third year, if the natural understorey vegetation is maintained, it will protect the soil from the raindrop impact and the subsequent surface crusting^9^. However, in the study area, understorey vegetation is systematically removed and/or burnt by the farmers, and soil is left bare and exposed to raindrop and overland flow (Fig. 6a,b and c). Consistently, F_OLF_ increases in correlation with the increase in Teak_>3_ (Table 1). Nevertheless, during the four last years of the second sub-period, overland flow slightly decreased with the increase of Fw&Fo (Fig. 2b). These observations are consistent with plot-scale measurements conducted in the same catchment, showing that infiltration rates under fallow are higher than those found under teak plantations^9^.

### Impact of land-use change on sediment yields

Sediment yields were significantly lower during the first sub-period (2002–2007) when the areal percentage of teak plantations was lower than 4%. However, a high SY value was observed in 2007 (833 Mg km^−^²). It was attributed to the particularly high areal percentage of annual crop observed during that specific year. Indeed, this land use is characterised by a low infiltrability due to the formation of soil surface crusting^9^, and a high sensitivity to overland flow and erosion^1^. From 2011 onwards, when Teak_>3_ (i.e. teak with degraded soil surface conditions) exceeds 20% each year, high SY and F_OLF_ values were observed (up to 1260 Mg km^−2^ and 44%, respectively, Fig. 2e). These high values cannot be related to the areal percentage of annual crops, which decreased from 40 to 4% during the second sub-period. Rainfall has long been known as a major control factor for soil erosion at the plot scale^32^, possibly explaining the high SY values observed after 2011. Indeed, high annual rainfall depths were measured in 2011 (1884 mm) and 2013 (1524 mm). However, the SY value measured in 2004 (232 Mg km^−2^) is lower than that of 2012 (609 Mg km^−2^), despite a 23% higher rainfall depth (978 mm and 1305 mm, respectively) and a similar areal percentage of annual crops (29% and 24% respectively). However, the areal percentage of Teak_>3_ increased from 2 to 18% between 2004 and 2012. Therefore, the most likely factor that may explain this difference is the increase in the areal percentage of teak plantations. This is demonstrated by the different behaviour observed in contrasted sub-catchments (S7 and S8; Fig. 3). A massive soil loss (2499 Mg km^−2^) occurred in the sub-catchment dominated by degraded teak plantations (without understorey) in 2014, whereas this production remained very low (127 Mg km^−2^) for the sub-catchment dominated by the traditional shifting cultivation (Fig. 3c). The difference is particularly visible during the most intense event of the period (i.e. 16 September 2014; Fig. 3a; 187 mm d^−1^; I_max_ = 144 mm h^−1^ based on 6-min measurements).

Since sediment yield is best explained and positively correlated to Rain_F and F_OLF_ (Fig. 4b) and F_OLF_ is best explained and positively correlated to Teak_>3_ and AC (Fig. 4a), it is consistent that the higher the surface covered with fallow and secondary forest, the lower is the sediment yield. In contrast, the higher the surface covered with teak plantations and annual crops, the higher is the soil loss.

Whatever the period considered, subsurface flow was the major contributor to stream discharge during floods. This process can lead to the more frequent collapse of channel banks and the remobilisation of material deposited in the channel^33^. Groundwater outflow can also decrease the concentration of suspended sediments in the river^16, 29^.

Measurements of ^137^Cs activities showed that soil erosion was so intense under teak plantations that the content of their soil surface in ^137^Cs decreased to reach values measured in subsurface material. This trend is reflected by the decrease in ^137^Cs activities with the increasing age of the teak plantations, revealing the major role of erosion in removing surface soil layers through the increased generation of rills and gullies^12^. This result is further supported by the fact that teaks were preferentially planted in the most accessible zones from the village, i.e. in areas with the deepest and most fertile soils.

### Synthesis and recommendations for future land-use management in Southeastern Asia

**Figure 7:**
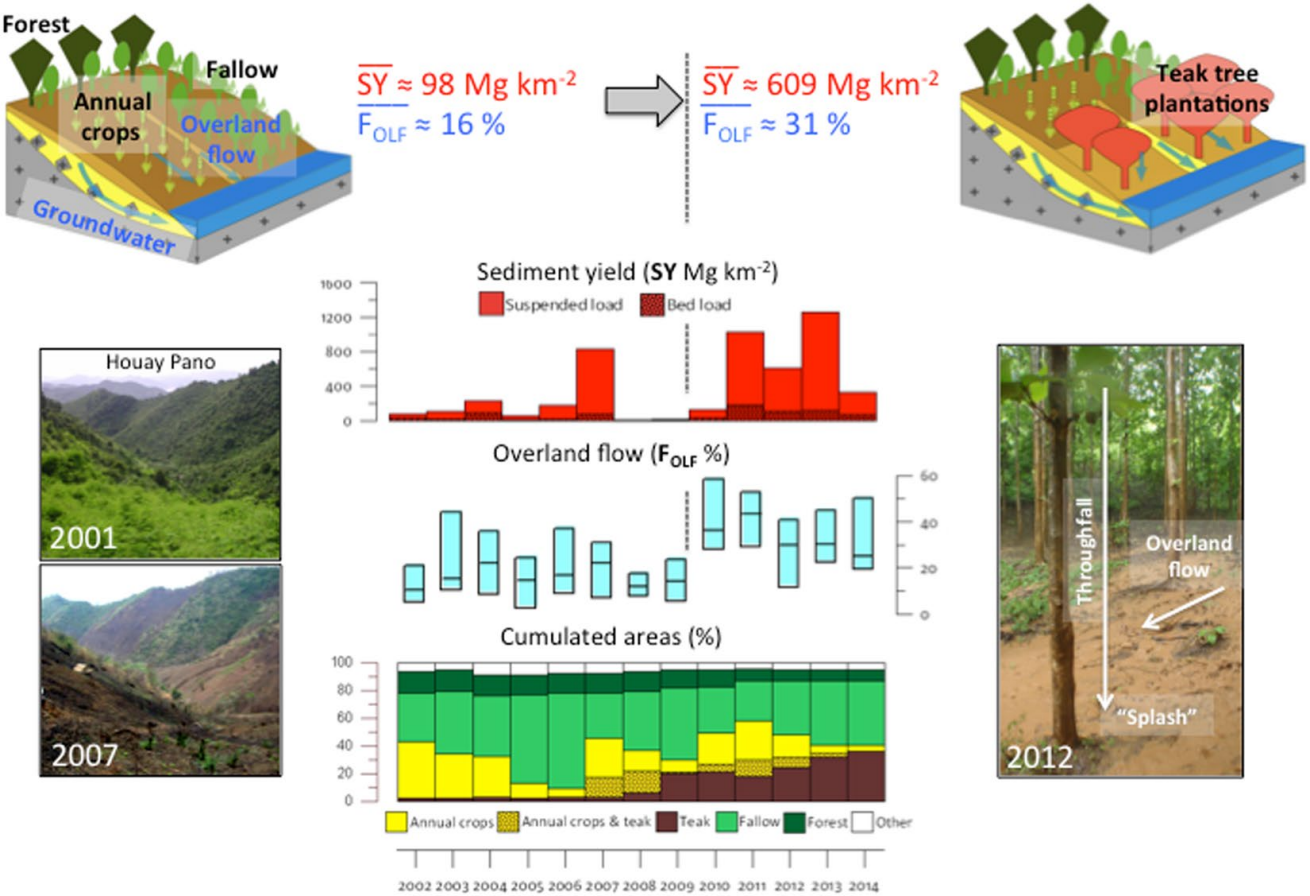
Graphical abstract: bottom left side, two pictures of the Houay Pano catchment in 2001 (almost entirely covered by fallow) and 2007 (after burning and before cropping); bottom right side, a picture of overland flow in a teak tree plantation during a rainfall event; centre, the figure shows plots of measurements conducted at the S4 monitoring station: annual sediment yield (SY) including suspended load and bed load contributions, box plots of overland flow contribution (F_OLF_) during flood events, and areal percentages of the catchment covered by the main land uses; top left and right, conceptual diagrams of the extreme land use situations observed in the catchment; top centre: inter-annual median values of SY and F_OLF_ for the two periods 2002–2009 (left) and 2010–2014 (right).

In Southeastern Asia, incentives to reduce shifting cultivation have induced rapid land-use changes, leading to a dramatic reduction in the cropland area and to a shortening of fallow periods^34^, which counter-productively resulted in an increase in land degradation and soil erosion as well as in a decline of crop yields^35, 36^. The lack of sustainability of these transitional farming systems^37^ has prompted the need for developing alternative production systems. These include perennial and monoculture tree plantations such as rubber (*Hevea brasiliensis*, *Muell. Arg*.) and teak (*Tectona grandis, L*.), which are rapidly expanding throughout montane environments^23^, as observed in the Houay Pano catchment (Fig. 7).

Most studies that investigated the impact of afforestation in montane cultivated catchments have demonstrated a reduction of soil losses at the hillslope level^38^. This trend is attributed to rainfall interception by the canopy, and to a reduction of the throughfall kinetic energy and the resulting splash erosion as the soil is better protected by various strata of vegetation cover^39, 40^. Furthermore, under these conditions, the soil surface hydrodynamic properties (i.e. hydraulic conductivity) recovered, as a result of the intensification of biological activity^41^, improving infiltration and the vertical drainage of rainfall and thereby reducing soil losses due to overland flow.

In the region where the current research was conducted, teak tree afforestation induced an opposite hydro-sedimentary response (Fig. 7). Most farmers intentionally kept the soil bare under mature teak trees through the frequent burning of the understorey. This practice resulted from a mix of local beliefs and practical considerations including the supposed need for a reduced competition for soil water and nutrients, and an improved access to and a better circulation within plantations. The soil surface in these managed teak tree plantations is exposed to raindrops having a kinetic energy exceeding that recorded in non-intercepted rainfall; the reason for this high kinetic energy is that the large leaves of teak intercept and concentrate rain drops which subsequently fall from an effective height exceeding the threshold (8 m) above which velocity no longer increases with height due to air resistance^42^. Besides detachment, exposition of soil to the splash process generates the formation of crusts (Fig. 6c) and the progressive clogging of soil porosity. Because of these two processes, surface soil structure is destroyed and soil surface becomes impermeable, which limits subsurface flow and intensifies overland flow, increasing the generation of rill and gully erosion occurring on these steep slopes. Moreover, reforestation with teak may also decrease baseflow due to the increased root water uptake and evapotranspiration^43^.

Based on the log-linear and linear relationships found in this current research (Fig. 4a,b), overland flow and total sediment yield by 2020 were estimated under the following scenario: (i) an expansion rate of teak tree plantations similar to that observed during the second sub-period (2008–2014) with teak plantations covering 75% of the catchment area by 2020; (ii) an unchanged fractional cover of annual crops and (iii) the stability of triggering-flood annual rainfall depth at a value corresponding to the average measured during the entire study period. Under these conditions, the statistical relationships predicted an increase of overland flow contribution during floods, from 28% in 2014 to approximately 40% in 2020, with a concomitant tremendous increase in annual sediment yield, from 429 Mg km^−2^year^−1^ in 2014 up to approximately 600 Mg km^−2^ year^−1^ in 2020, which exceeds by far the soil loss rate of 250 Mg km^−2^year^−1^ considered by most soil scientists as the highest tolerable at the plot scale^44^, and which generally exceeds by far soil loss rates observed at the catchment scale.

As a recommendation, removal of ground vegetation regrowth and burning of the understorey vegetation under teak tree plantations should be strongly discouraged to avoid the degradation of the soil surface (soil surface crusting) and the subsequent increased generation of overland flow and soil loss. Alternatively, planting a shade-tolerant crop as an understorey potentially providing some economic return could be tested; candidate species include ginger (*Zingiber officinale)*, cardamom (*Elettaria cardamomum*), sweet potato *(Ipomoea batatas)* or broom grass (*Thysanolaena maxima*). However, improper harvesting practices of these tuber or rhizome crops may also generate severe soil erosion. One promising avenue to improve the system is to train the farmers to forestry with lower planting densities without necessarily reducing the timber yield per surface unit.

## Methods

### Study site and land uses

The Houay Pano catchment (60 ha) is located 10 km south of Luang Prabang in northern Laos, in the Mekong River basin (Fig. 1). Mean annual temperature is 25.3 °C^10^.The tropical monsoon climate of the region is characterized by the succession of dry and wet seasons, with 80% of annual rainfall occurring from May to October^45^. Water and sediment fluxes were monitored at the outlet of the 60-ha catchment (Fig. 1a)^1^ since 2001 (at the so-called S4 station). In addition, during the rainy season in 2014, fluxes were monitored at the outlet of two zero-order headwater nested catchments (i.e. hollow valleys, so-called S7 and S8 stations). Soils consist of deep ( > 2 m) and moderately deep ( > 0.5 m) Alfisols, except along crests and ridges where Inceptisols are found^46^. The main land uses in the area are annual crops (upland rice and Job’s tear), fallow, teak plantations and secondary forests^8, 47^. Their respective dominance in the catchment changed during the last decade: the slash-and-burn cultivation system that dominated between 2002 and 2007 has gradually been replaced with teak tree plantations^48^ (Fig. 2b). Detailed land-use surveys and mapping were conducted each year. The areal percentages of each land use (±10%) in the catchment were calculated using QGis (http://www.qgis.org/). S7 and S8 sub-catchments have similar soil characteristics (Fig. 1a), surface areas (0.60 and 0.57 ha) and mean slope gradients (62 and 54%) although, during the monitoring year 2014, they greatly varied in land use. In S7, the main land uses were teak monoculture with an abundant understorey preserved from burning (22% of surface area), and typical shifting cultivation mosaics (78% of the surface including upland rice 23%, Job’s tears 14%, fallow 34%, secondary forest 7%). In contrast, the S8 sub-catchment was covered with teak monoculture with a degraded soil surface in lower parts (42% of the total surface), and secondary forest (58%) in upper parts.

### Hydro-sedimentary monitoring

Rainfall was measured every 6-min using 1 automatic tipping-bucket gauge and 6 manual gauges across the catchment (Fig. 1). The maximal spatial variability of annual rainfall is estimated at 20%. Stream discharge was calculated from the continuous monitoring of the water level since 2002. Water level was recorded with a OTT Thalimedes device after every 0.5 cm variation. Uncertainties on river discharge are estimated to be below 10%. Suspended sediment was collected using an automatic sampler (Fig. 6e) during each flood event during the 2002–2014 period (samples were collected after every 2-cm water level variations during the rising stage and after every 5-cm water level variations during the falling stage). Bedload sediment was estimated each month using a sediment trap (Fig. 6e). Suspended and bed load sediments were estimated following the approach described by Valentin *et al*.^1^. An average of 20 river water (600 mL) samples was collected ~10 cm below the river surface during each flood event. Overland flow was estimated based on electrical conductivity^6^ measured in these samples. Infiltration rates were estimated by calculating the difference between rainfall and overland flow divided by the duration of the rainfall event. Total sediment fluxes correspond to the export of both bedload and suspended sediment, and these values are provided with 10% uncertainties^1^. All hydro-sedimentary data are available on-line (http://msec.obs-mip.fr/).

### Soil and sediment sampling

Soil surface samples (top 0–5 cm) were collected with 100-cm^3^ soil density cylinders along toposequences^8^ between 2000 and 2005 (*n* = 55) and in 2012 (*n* = 32) to characterize potential sources of sediment (Fig. 1)^29^. In addition, gully and channel bank samples (*n* = 23) were collected in 2012 by scraping the material over the entire exposed surface of channel banks (up to 50 cm) and gullies (1–5 cm) to characterize subsurface sources (Fig. 1)^27^. In teak plantations, soil surface samples (top 0–5 cm, *n* = 35) were collected along toposequences by scraping soil, 20 cm upstream and downstream of trees, as well as between trees, under teak plantations of different ages (5, 19, 25 years). Samples were then regrouped for each type of plantation. Finally, a composite sediment sample representative of the total annual export at the catchment outlet (S4 station) was prepared for 2002 (dominance of slash-and-burn cultivation) and 2012 (period of expansion of teak plantations).

### Fallout radionuclide activities of the soil and sediment samples

Caesium-137 (^137^Cs) was measured at the Laboratoire des Sciences du Climat et de l’Environnement (Gif-sur-Yvette, France) following the methods detailed by Gourdin *et al*.^29^. All results are expressed in Bq kg^−1^ with all activities decay-corrected to 2012. Uncertainties on radionuclide activities reached up to 30%, depending on the counting time and the quantity of material available for analysis.

### Statistical analyses

Multiple regressions were computed to determine linear or log-linear relationships^49, 50^ between the independent variables calculated for each year, namely contribution of overland flow during floods (F_OLF_, annual median percentage of total stormflow) and total annual soil loss (SY), and several candidate explanatory variables calculated for each year (i.e. Rain, Rain_F, OLF, F_OLF_, InfRate, SL, BL, SY, Fw&Fo, AC, Teak, Teak_>3_). To predict each independent variable, the selection of the best set of explanatory variables was guided by ‘best subsets’ and ‘step-wise’ regressions, two selection algorithms –available in the Minitab 17 free trial statistical packages (https://www.minitab.com/en-us/products/minitab/) – that intend to maximize the prediction R-squared (R^2^_pred_) calculated by leave-one-out cross-validations. R^2^_pred_ reflects the ability of the model to predict observations which were not used in the model calibration. An explanatory variable was considered to be statistically significantly different from zero when its *p*-value, derived from the Student’s *t*-test, was lower than 0.05. The required homoscedasticity of the model residuals was verified by visual inspection. Possible multi-collinearity among the explanatory variables was controlled with the variance inflation factor^51^.

The Tukey-Kramer Honestly Significant Difference (HSV) test was applied to compare ^137^Cs activities measured in various soil and sediment sample groups, after rejection of the ANOVA null hypothesis of equal means (Table 2).
